# Manipulating the In Vivo Behaviour of ^68^Ga with Tris(Hydroxypyridinone) Chelators: Pretargeting and Blood Clearance

**DOI:** 10.3390/ijms21041496

**Published:** 2020-02-22

**Authors:** Cinzia Imberti, Pierre Adumeau, Julia E. Blower, Fahad Al Salemee, Julia Baguña Torres, Jason S. Lewis, Brian M. Zeglis, Samantha Y. A. Terry, Philip J. Blower

**Affiliations:** 1 School of Biomedical Engineering and Imaging Sciences, King’s College London, Fourth Floor Lambeth Wing, St Thomas’ Hospital, London SE1 7EH, UK; 2 Department of Chemistry, Hunter College, City University of New York, New York, NY 10021, USA; 3 Department of Radiology, Memorial Sloan Kettering Cancer Center, New York, NY 10065, USA

**Keywords:** metal chelation, radionuclide imaging, pretargeting, gallium-68, hydroxypyridinones, monoclonal antibodies, bifunctional chelators

## Abstract

Pretargeting is widely explored in immunoPET as a strategy to reduce radiation exposure of non-target organs and allow the use of short-lived radionuclides that would not otherwise be compatible with the slow pharmacokinetic profiles of antibodies. Here we investigate a pretargeting strategy based on gallium-68 and the chelator THP^Me^ as a high-affinity pair capable of combining in vivo. After confirming the ability of THP^Me^ to bind ^68^Ga in vivo at low concentrations, the bifunctional THP^Me^-NCS was conjugated to a humanised huA33 antibody targeting the A33 glycoprotein. Imaging experiments performed in nude mice bearing A33-positive SW1222 colorectal cancer xenografts compared pretargeting (100 μg of THP^Me^-NCS-huA33, followed after 24 h by 8–10 MBq of ^68^Ga^3+^) with both a directly labelled radioimmunoconjugate (^89^Zr-DFO-NCS-huA33, 88 μg, 7 MBq) and a ^68^Ga-only negative control (8–10 MBq of ^68^Ga^3+^). Imaging was performed 25 h after antibody administration (1 h after ^68^Ga^3+^ administration for negative control). No difference between pretargeting and the negative control was observed, suggesting that pretargeting via metal chelation is not feasible using this model. However, significant accumulation of “unchelated” ^68^Ga^3+^ in the tumour was found (12.9 %ID/g) even without prior administration of THP^Me^-NCS-huA33, though tumour-to-background contrast was impaired by residual activity in the blood. Therefore, the ^68^Ga-only experiment was repeated using THP^Me^ (20 μg, 1 h after ^68^Ga^3+^ administration) to clear circulating ^68^Ga^3+^, producing a three-fold improvement of the tumour-to-blood activity concentration ratio. Although preliminary, these results highlight the potential of THP^Me^ as a ^68^Ga clearing agent in imaging applications with gallium citrate.

## 1. Introduction

Monoclonal antibodies display exquisite targeting properties and have dramatically changed the way we diagnose and treat cancer. Labelling of antibodies with radiometals for imaging and radionuclide therapy traditionally requires antibody conjugation to a bifunctional chelator (BFC) able to bind covalently to an amino acid residue or glycan side-chain at one end and to efficiently coordinate the radiometal at the other. Examples of FDA-approved radiolabelled antibodies include the ^90^Y-labelled Ibritumomab tiuxetan for the radioimmunotherapy of B-cell non-Hodgkin lymphoma [1] and the ^111^In-labelled capromab pendetide for prostate cancer radioimmunoscintigraphy [2]. The concept has been extended in recent years to include radioimmunoPET by virtue of long-lived positron-emitting radionuclides such as zirconium-89 [3].

One major limitation of radiolabelled antibodies is slow clearance arising from their large size (approximately 150 KDa, compared to ≤1 KDa for the radiolabelled BFC) and lack of domains that facilitate clearance by molecular recognition pathways. Together these result in longer blood half-life and slower extravasation and tissue penetration compared to the radiolabelled chelator on its own. Hence, the antibody circulates in the blood for days (48 h or even more) before reaching an acceptable tumour-to-blood ratio, leading to delayed imaging and prolonged radiation exposure of healthy tissues. In addition, this limits the choice of radiometals that can be used for imaging with antibodies to those possessing a long half-life comparable with the blood half-life of the immunoconjugate so that the activity has not decayed by the time sufficient contrast is achieved.

Pretargeting provides a unique approach to tackle this issue, by separating the inevitably slow targeting/clearance of the antibody from the delivery of the radioactive probe, which, owing to its smaller size, can be considerably quicker (minutes to hours). For a pretargeting approach to be successful, these two components should have great affinity for one another and very fast (preferably diffusion-limited) chemical association so that they are able to combine in vivo, at the tumour site, to give the fully functional radiolabelled antibody [4].

Some pretargeting strategies that have been successfully investigated and translated into clinical trials have used bispecific antibodies/haptens or biotin/avidin as the high-affinity chemical pair [5]. Other promising approaches are based on complementary oligonucleotide pairs, and bio-orthogonal click chemistry [6]. Notably, the common feature of all these strategies is that part of the connecting moiety is incorporated into the antibody and the other (chelator) part is radiolabelled separately. This radiolabelled probe is administered at a later stage when the excess circulating antibody has cleared from the blood. The optimal time between the two administrations can be estimated by imaging with a directly radiolabelled antibody.

**Figure 1:**
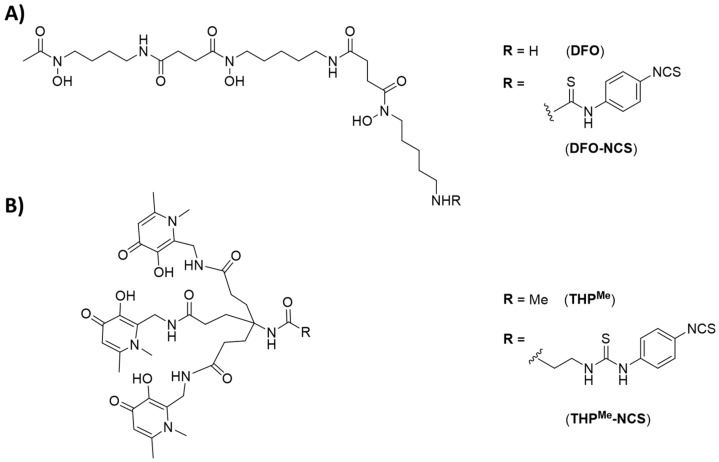
Chemical structures of the chelators used in this work. (**A**) Bifunctional chelator (DFO) and its bifunctional analogue DFO-NCS, (**B**) THP^Me^ and THP^Me^-NCS.

We previously demonstrated that the tris(hydroxypyridinone) chelator THP^Me^ (Figure 1A) and its second-generation analogue THP^H^ possess high thermodynamic affinity for the short-lived radiometal ^68^Ga (pGa = 30.0 for THP^Me^ at physiological pH) [7,8] and were able to complex ^68^Ga very rapidly at neutral pH even at very low chelator concentration. Notably, THP^Me^ was able to bind ^68^Ga in serum, and the radiolabelled complex [^68^Ga(THP^Me^)] is stable in the presence of apotransferrin [7,9]. These outstanding radiolabelling properties, already exploited as a basis for rapid, one-step kit-based ^68^Ga radiolabelling of both small-molecule and protein-based PET radiopharmaceuticals [10,11,12], allow them to bind ^68^Ga^3+^ in vivo, when administered i.v. to mice previously injected with ^68^Ga (as confirmed by radioHPLC analysis of the urine) despite the in vivo conditions of extreme dilution and competing affinities [8]. Thus, ^68^Ga^3+^ and THP^Me^ (or THP^H^) represent a potential chemical pretargeting pair. Notably, several BFC have been developed based on THP^Me^, including isothiocyanate (THP^Me^-NCS, Figure 1A) and maleimide derivatives that can effectively bind to lysine or cysteine side chains, respectively, in mild conditions (aqueous solution, mildly basic pH, room temperature), allowing facile conjugation to antibodies [10,13,14].

**Figure 2:**
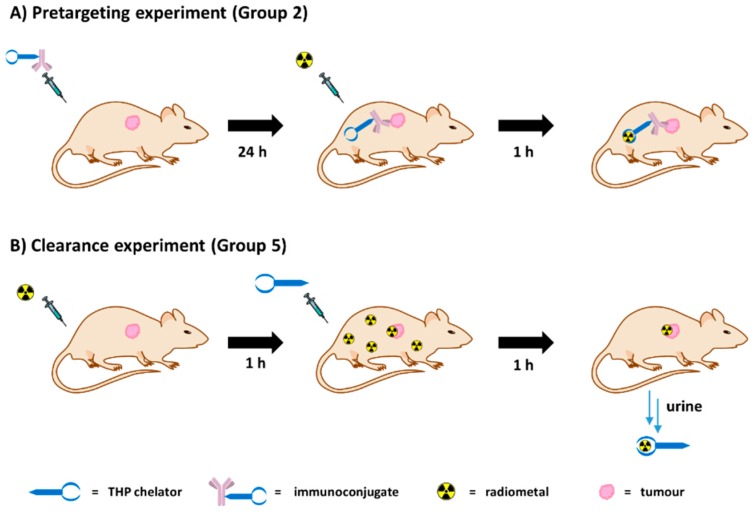
Schematic representation of the experimental workflow for the reported pretargeting experiment (**A**) and clearance experiment (**B**) using ^68^Ga and THP^Me^ chelators.

We therefore hypothesised that a different pretargeting strategy, based on metal chelation, could be feasible using a radiometal (^68^Ga^3+^) and a BFC (THP^Me^-NCS, Figure 1A) as a high-affinity chemical pair. In this pretargeting approach, the whole BFC would be attached to the antibody and then radiolabelled in vivo at the tumour site as schematically represented in Figure 2A. This strategy would minimise handling of the radioactivity, compared to traditional pretargeting approaches, since no radiosynthetic work is required other than buffering the radiometal. This would in turn reduce operators’ exposure and greatly simplify the clinical protocol so that it could be performed in hospitals lacking extensive radiochemistry facilities and expertise.

Herein, we tested the pretargeting by metal chelation hypothesis in a murine xenograft model of human colorectal cancer (SW1222) utilising THP^Me-^NCS-conjugated huA33 antibody as a pretargeting vector and acetate-buffered ^68^Ga as the radioactive species. The transmembrane glycoprotein A33 targeted by huA33 is expressed in 95% of colorectal carcinomas [15] (including the colorectal cancer cell line SW1222 used in this work). It remains on the cell surface upon binding to the antibody and is not internalised or shed. Therefore, this antibody/antigen couple represents an ideal model for pretargeting, as already demonstrated in preclinical studies of the bio-orthogonal click chemistry pretargeting approach [16,17,18]. We also evaluated the potential of THP^Me^ as a means of quickly removing background radioactivity after administration of ^68^Ga^3+^ salts, which similarly relies on efficient in vivo chelation of ^68^Ga^3+^ by THP^Me^ (Figure 2B).

## 2. Results

DFO is an FDA-approved treatment for iron overload [19] and had also been investigated in the past as a blood-clearance agent for ^67^Ga imaging, owing to the similarity between high-spin Fe(III) and Ga(III) [20,21]. In competition studies in vitro, THP^Me^ was shown to out-compete DFO for binding to ^68^Ga^3+^ [22]. Nevertheless, it was important to investigate, prior to undertaking pretargeting studies, whether DFO would be a better candidate than THP^Me^ to test the pretargeting via metal chelation hypothesis in vivo. The in vivo Ga(III) binding abilities of THP^Me^ and DFO were compared by injecting mice with each of these chelators in an equimolar dose (24 nmol), or saline as a negative control, 1 h after ^68^Ga^3+^ administration.

**Figure 3:**
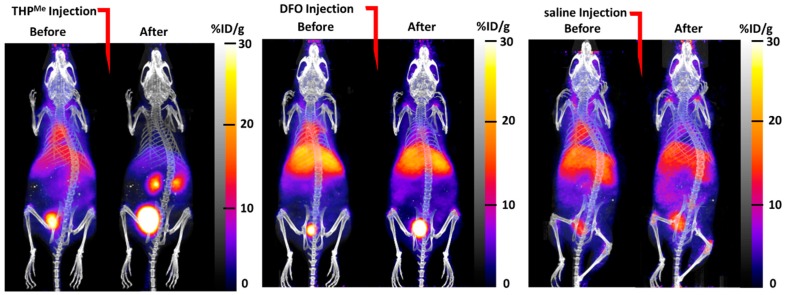
PET/CT maximum intensity projection (MIP) images of healthy mice injected with ^68^Ga-acetate, followed at 1 h post injection by treatment with 24 nmol of THP^Me^ (left pair of images), DFO (middle pair) or saline (right pair) as negative control (voxel size 0.21 × 0.21 × 0.21 mm^3^).

The maximum intensity projection (MIP) images (Figure 3) show how biodistribution of the activity was markedly affected by injection of THP^Me^ compared to saline: there was a sudden and dramatic increase in renal clearance, while the effect of DFO was less visible. When comparing ex vivo biodistribution, THP^Me^ showed a statistically significant increase in ^68^Ga accumulation in kidneys compared to saline (%ID/g = 112 ± 43 for THP^Me^ treated mice vs. 6.2 ± 2.4 for saline group), while no such effect was visible for DFO (%ID/g in kidneys = 6.7 ± 2.9). On the other hand, DFO caused a reduced and less variable gallium accumulation in femur and liver (%ID/g in femur = 6.8 ± 1.2, liver = 17.5 ± 7.6) compared to the control group (%ID/g in femur = 15 ± 11, liver = 22 ± 19), although to a lesser extent than for THP^Me^ (%ID/g in femur = 2.8 ± 0.5, liver = 12.5 ± 3.0) (Figure S1). Even though no statistically significant difference was detected between the chelators and control groups for these organs, likely due to the large variability observed for the saline group, these data suggest that DFO is also, to some extent, able to bind circulating ^68^Ga under these conditions.

Overall, these results support our hypothesis that THP^Me^ is equal or superior to DFO in binding ^68^Ga^3+^ effectively in vivo and clearing it renally, and suggest that THP^Me^ would be a promising candidate for testing our pretargeting via metal-chelation approach.

A dose-finding study was also conducted to verify that THP^Me^ maintained its ability to bind ^68^Ga in vivo even when its concentration was significantly reduced. Mice were injected with ^68^Ga and then with different concentrations of the chelator. Notably, a significantly different ^68^Ga biodistribution compared to the saline-only control was observed even for the lowest dose considered of THP^Me^ (1 μg, 1.2 nmol), and almost no difference was found between the 5 μg (6 nmol) and 20 μg (24 nmol) groups for most organs (Figure S2). This indicated that THP^Me^ radiolabelling in vivo was effective even at low micromolar concentration of chelator, suggesting that radiolabelling of a pretargeted tumour would be feasible.

### 2.1. Immunoconjugate Characterisation and Radiolabelling

Conjugation of THP^Me^-NCS to the huA33 was initially performed using 5–10 molar equivalents of bifunctional chelator, following modified published procedures [23]. Conjugation yield was calculated based on measurements of the antibody concentration after immunoconjugate purification via size exclusion chromatography (Table S1). Matrix-assisted laser desorption-ionisation (MALDI) analysis of the immunoconjugates compared to the native antibodies (Figure S3) allowed us to determine average chelator-to-antibody ratios (degree of labelling, DOL) of 0.6 and 0.4 achieved for the 10 and 5 molar equivalents batches, respectively (Table S1).

Radiolabelling of the THP^Me^-NCS-huA33 conjugate was initially performed at a slightly acidic pH (pH 5.5–6, as measured using pH paper) obtained by neutralisation of the generator eluate ^68^Ga with ammonium acetate buffer, as previously reported for this chelator [7,13]. The radiochemical yield of the crude product was measured by ITLC using 50 mM EDTA as a mobile phase (Figure S4). In addition, the radiolabelled immunoconjugate was run through a PD10 size exclusion column to rule out the presence of scarcely soluble Ga(OH)_3_ species, which would not have been revealed under the ITLC conditions utilised, but would be trapped on the size exclusion column. Quantitative radiolabelling was obtained for both 5 molar equivalents and 10 molar equivalents batches at antibody concentrations as low as 1 μM, confirming successful conjugation. Under the same conditions the native antibody did not show radiolabelling, confirming that the THP^Me^- NCS chelator is necessary for efficient ^68^Ga binding to the antibody.

Radiolabelling of the immunoconjugate was also investigated in conditions that more closely resemble those of preclinical experiments. Neutral pH is desirable for intravenous injection in conscious mice and the pH experienced by the radiometal in vivo is also close to neutral. Therefore, the effect of eluate neutralisation to pH 7–7.2 on radiolabelling was evaluated. In addition, to take into account the time interval between eluate neutralisation and injection of the mice, the effect of a 45 min delay between eluate neutralisation and radiolabelling of the 10 equivalent batch was investigated (Figure S5). Remarkably, even in this case radiolabelling was quantitative with radiochemical yield >95%, consistent with previous reports of highly efficient neutral-pH labelling of proteins conjugated with THP^Me^ [10].

Finally, to verify that conjugation to the chelator did not impair the antibody’s ability to bind to its target receptor, the immunoreactive fraction was determined for both the 5 and 10 molar equivalents batches via an immunoreactivity assay (Table S1) [24]. In this experiment, a large excess of antigen is employed, able to bind the totality of the added radioimmunoconjugate (≈ 100% uptake) if its immunoreactivity is undiminished compared to the unmodified antibody. Accordingly, the observed uptake percentage reflects the immunoreactive fraction of the radioimmunoconjugate batch. For both the batches used in pretargeting experiments, only marginal detriment of the immunoreactivity was found, with immunoreactivity fractions equal or superior to 90%. Based on these results, the 10 molar equivalents batch was selected to perform preclinical experiments.

### 2.2. In Vivo Pretargeting Experiments

Preclinical experiments were performed in nude mice bearing human SW1222 colon cancer xenografts. Different groups, of five mice each, were considered (Table 1), as discussed below.

Notably, the use of directly radiolabelled ^68^Ga-THP^Me^-NCS-huA33 as a positive control was precluded by the short half-life of the radiometal. Therefore, prior to conducting pretargeting experiments using ^68^Ga and THP^Me^-NCS-huA33, PET imaging of the same tumour model was undertaken with directly labelled ^89^Zr-DFO-NCS-huA33 immunoconjugate, to verify that the xenografts expressed the A33 antigen targeted by the huA33 antibody and that 24 h was sufficient time for the radioimmunoconjugate to localise at the tumour site and clear from circulation.

**Figure 4:**
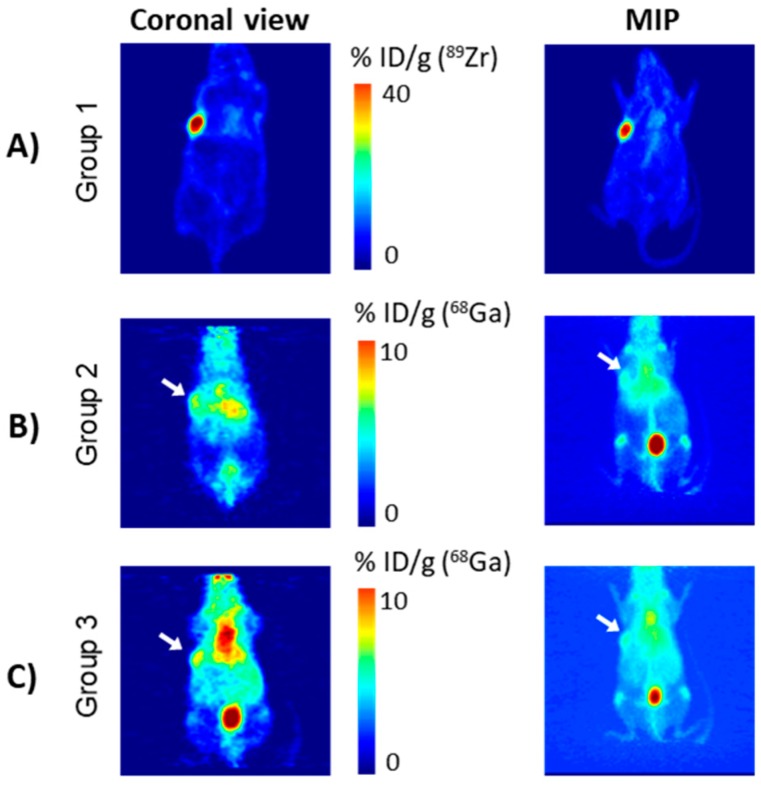
Representative PET images of nude mice bearing SW1222 xenografts. (**A**) Mouse treated with directly labelled ^89^Zr-DFO-NCS-huA33 (25 h after injection, group 1); (**B**) mouse pretreated with THP^Me^-NCS-huA33 (group 2) followed at 24 h by ^68^Ga acetate (imaged at 2 h 10 min after ^68^Ga injection); (**C**) mouse treated with ^68^Ga acetate only (2 h 30 min after ^68^Ga injection, group 3). Images are reported as coronal section (left panel, thickness 0.87 mm) and MIP (right panel), voxel size is 0.87 × 0.87 × 0.80 mm^3^. White arrows indicate location of the tumour.

Radiolabelling of the DFO-NCS-huA33 immunoconjugate with ^89^Zr was achieved in nearly quantitative yield and purity, as confirmed by ITLC analysis of the fractions eluted from post-radiolabelling size-exclusion chromatography columns (Figure S6). Imaging of mice injected with ^89^Zr-labelled DFO-NCS-huA33 immunoconjugate (group 1), performed at 25 h following radioimmunoconjugate administration, showed selective tumour localisation of the radioimmunoconjugate (Figure 4A).

**Figure 5:**
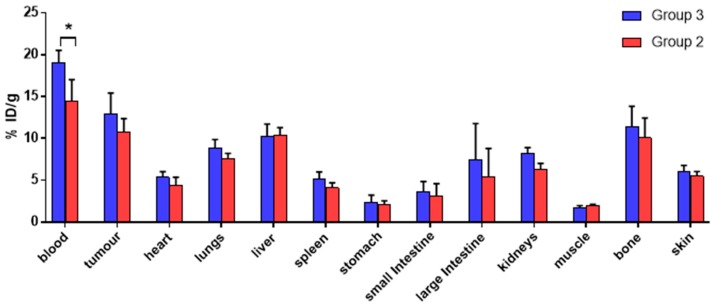
Ex vivo ^68^Ga biodistribution in nude mice bearing SW1222 xenografts at 3 h after ^68^Ga injection, comparing mice pretreated with THP^Me^-NCS-huA33 (group 2) 24 h before ^68^Ga injection (red), with mice that were not pretreated with any immunoconjugate (group 3, blue). For each group *n* = 5, data are presented as mean ± SD. Comparison between the two groups was performed by a two-tailed *t*-test for each organ (* *p* < 0.05).

Mice in group 2 (the pretargeting group) were pretreated with 100 μg of the THP^Me^-NCS-huA33 conjugate (10 eq. batch) followed, after 24 h, by acetate-buffered ^68^Ga (pH ≈ 7) generator eluate. To ensure uniformity in the Ga^3+^ speciation at the time of injection, all mice were injected with ^68^Ga at the same time (10 min range). Therefore, imaging of the mice had to be staggered with respect to the injection times, with the first mouse scanned at 50 min after the injection of ^68^Ga and the last scanned at 90 min after injection. The scanning was then repeated after one further hour to investigate the effect of a longer delay between ^68^Ga injection and PET imaging on image quality and contrast. A representative PET scan at 130 min after ^68^Ga administration is shown in Figure 4B; the 70 min time point for the same mouse and a representative PET scan for a second mouse are also reported in the supplementary information (Figure S7). Ex vivo biodistribution was then determined at 3 h after ^68^Ga injection and is reported in Figure 5 (red histograms). Both the PET scans and the ex vivo biodistribution data showed some accumulation of ^68^Ga in the tumour (≈11 %ID/g). However, the tissue with the highest radioactivity concentration was blood even at 3 h after the ^68^Ga injection. Uptake in non-target organs, such as liver and bones (typical of the in vivo behaviour of unchelated gallium [25]), was also visible.

As a negative control, five mice (group 3) were injected with acetate buffered ^68^Ga, without prior injection of antibody-chelator conjugate. Imaging and ex vivo biodistribution were performed as in the pretargeting group (group 2) and, notably, showed very similar ^68^Ga biodistribution to those in group 2, as evident in the PET images (Figure 4C and Figure S8).

A comparison of the ex vivo biodistribution of ^68^Ga in groups 2 and 3 (Figure 5, *t*-test performed for each organ) also revealed no significant difference in tumour ^68^Ga uptake for the two groups (10.8 ± 1.5 %ID/g in group 2 vs. 12.9 ± 2.5 %ID/g in group 3). The only significant difference among the organs examined was found for blood, whose uptake was higher for group 3 (19.1 ± 1.4 %ID/g) compared to group 2 (14.4 ± 2.6 %ID/g). Overall, these results suggest that the observed activity accumulation in the tumour xenografts in group 2 is due to direct uptake of unchelated “free” ^68^Ga and is not mediated by antibody pretargeting and subsequent in vivo radiolabelling of the antibody-chelator conjugate.

As in vivo radiolabelling efficacy is expected to be highly dependent on local accessible chelator concentration, we hypothesised that failure to observe a pretargeting effect could be explained by insufficient local concentration of the chelator on the tumour surface. Therefore, in a second attempt at pretargeting we repeated the immunoconjugation with higher molar equivalents of chelator, aiming to increase the average chelator/antibody ratio of the conjugate and, hence, the concentration of chelator available at the tumour cell surface for in vivo radiolabelling. A first attempt at antibody-chelator conjugation using 50 molar equivalents of chelators resulted in precipitation of the immunoconjugate and was therefore abandoned; while an immunoconjugation performed using 30 equivalents of THP^Me^-NCS was successful. MALDI revealed a modest increase in the average chelator-to-antibody ratio of the resulting immunoconjugate from 0.6 (for the 10 molar equivalents batch) to 1.0, the highest ratio achievable without precipitation (Table S1). As a result, this new batch was utilised in a second pretargeting experiment. However, no significant difference was found in the tumour uptake comparing the 30 molar equivalents pretargeting group (group 4) and the group 3 controls (Figures S9 and S10). Instead, ANOVA analysis of the data revealed lower bone uptake for the pretargeting 30 eq. group (6.0 ± 1.1 %ID/g) compared to both the control group 3 (11.4 ± 2.4 %ID/g) and to group 2 (10.1 ± 2.4 %ID/g).

### 2.3. Exploiting the In Vivo Chelation Strategy to Increase Contrast

Although our initial pretargeting by metal chelation strategy proved unpromising in this experimental setup, it was interesting to notice the relatively high tumour accumulation of free gallium in this xenograft model (>10 %ID/g), comparable to values observed when targeting tumours with ^68^Ga-radiolabelled peptide (see for example [12]), although the tumour/blood ratio was nevertheless <1. Noting that our previous studies showed that the in vivo radiolabelling of THP^Me^ had a profound impact on the biodistribution of ^68^Ga^3+^ [8], significantly accelerating its renal clearance in the urine, we decided to evaluate the use of THP^Me^ as a contrast-enhancing agent in the same model. In this additional experiment, five mice (group 5) were administered acetate-buffered ^68^Ga followed, at 1 h post injection, by THP^Me^ (20 μg, 24 nmol), aiming to clear the radiometal selectively from blood, thus increasing the tumour-to-blood activity ratio.

**Figure 6:**
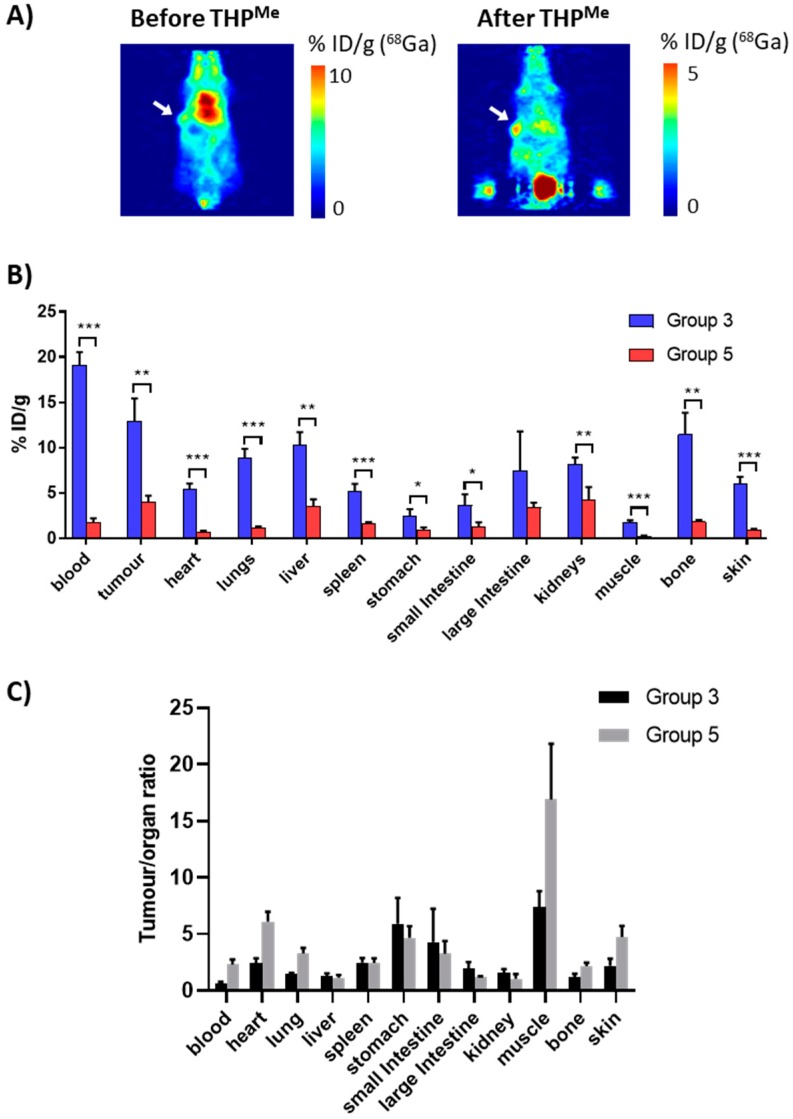
Investigation on the use of THP^Me^ as a ^68^Ga blood clearance agent. The upper panels (**A**) show example PET images (coronal view, thickness 0.87 mm) illustrating the biodistribution of ^68^Ga in a nude mouse bearing SW1222 xenografts before ((**left**), 40 min after ^68^Ga administration) and after ((**right**), 80 min after ^68^Ga administration) the injection of THP^Me^ as a blood clearance agent at 1 h after ^68^Ga administration. Note that the colour scales for the “before” and “after” images are different. Hot spots visible outside the mouse body in the coronal projection (**A, right panel**) are image artefacts due to the extremely high proportion of the activity in the bladder. White arrows indicate location of the tumour. Voxel size is 0.87 × 0.87 × 0.80 mm^3^. The middle panel (**B**) shows ex vivo ^68^Ga biodistribution at 3 h after ^68^Ga injections in nude mice bearing SW1222 xenografts, comparing mice treated with THP^Me^ at 1 h post ^68^Ga injections and the control group. For each group *n* = 5. Data are presented as mean ± SD. Comparison between the two groups was performed by a two-tailed *t*-test for each organ. * = *p* < 0.05, ** = *p* < 0.0001, *** = *p* < 10^−5^. The lower panel (**C**) compares tumour-to-organ ratios of %ID/g for treated and untreated mice, showing that treatment with THP^Me^ increases tumour-to-blood and other tumour-to-background ratios that are likely to affect tumour detection.

Mice were imaged from 20 min after THP^Me^ injection, with two of them also imaged between the ^68^Ga and the THP^Me^ injection, in order to compare the PET images before and after THP^Me^-mediated blood clearance in the same animal. An exemplar PET image is presented in Figure 6, together with the ex vivo biodistribution (3 h after ^68^Ga injection) compared to the ^68^Ga control group (group 3). Calculated *p*-values and tumour/organ ratios for each organ are reported in Table S2.

Notably, both PET images and ex vivo biodistribution data revealed the dramatic effect of THP^Me^ injection, which significantly decreased the uptake of ^68^Ga in most organs. A *t*-test revealed significantly reduced uptake in all organs except the large intestine, probably as a result of the larger variability in the %ID/g for this organ. Interestingly, the clearance effect is far more noticeable for the blood than for the tumour and many other tissues (Figure 6B); the tumour-to-blood ratio increased from 0.7 in the ^68^Ga-only control to 2.3 in the blood clearance experiment (> threefold increase) and similarly the tumour-to-muscle ratio from 7.4 to 16.9 (Figure 6C).

## 3. Discussion

In this work we performed an in vivo investigation of a new pretargeting hypothesis based on metal chelation, where the high-affinity chemical pair is represented by a radiometal and a chelator anchored to the antibody. The in vivo biodistribution of radiolabelled antibody ^89^Zr-DFO-NCS-huA33, whose ability to target A33 has been extensively validated [17], was used here as a positive control. This experiment confirmed that the xenografts expressed the desired glycoprotein and that the batch of huA33 antibody used in this work was able to selectively accumulate in the tumour tissue, and to do so in the 25 h between injection of the antibody and imaging, in agreement with previous findings [17]. The radiometal-chelator chemical pair utilised for the pretargeting studies consisted of ^68^Ga^3+^, administered in an acetate buffered solution, and the THP^Me^-based chelator THP^Me^-NCS coupled to the antibody [7,13]. The ability of THP^Me^ to capture ^68^Ga^3+^ in vivo has been previously reported and was corroborated here with a more thorough examination [8], including a comparison with the clinically established iron chelator DFO. This highlighted the superior Ga^3+^ binding ability of THP^Me^, while a dose-finding study confirmed successful in vivo chelation of ^68^Ga(III) even at a very small (1.2 nmol) dose of chelator.

Amongst the several available bifunctional derivatives of THP^Me^ [7,13], for our pretargeting studies we decided to use the isothiocyanate derivative THP^Me^-NCS [13], which can non-specifically bind to lysine residues in the antibody. This approach was preferred to site-specific conjugation because it allows, in principle, the attachment of a higher number of chelators to the antibody. In addition, previous pretargeting studies found no improvement in image contrast and quality when site-specific rather than non-site-specific conjugation was used in the same animal model [16].

Conjugation of the selected antibody was initially performed using 5 and 10 molar equivalents of THP^Me^-NCS and its success was demonstrated by efficient ^68^Ga radiolabelling of the purified immunoconjugates, but not of the native antibody, in the mild conditions typically used for ^68^Ga labelling of THP^Me^ bioconjugates [7,13]. The modified antibody was radiolabelled quantitatively, without need of purification, at neutral pH and immunoconjugate concentration as low as 1 μM. Moreover, the radiolabelling efficiency was maintained after pre-neutralisation of the eluate 45 min before radiolabelling—conditions that mimic the conditions of the in vivo pretargeting experiment and that often impair radiolabelling when other chelators are used. Testing under these conditions was important to rule out any effect of different gallium speciation (e.g., colloid formation) over the pH range or time when considering the results of preclinical experiments. In fact, in our experience (unpublished results), unchelated gallium often displays a variable in vivo behaviour after the pH has been raised above 3, presumably owing to the multitude of species, including potentially colloidal gallium hydroxides, that may be present in these conditions. An immunoreactivity assay also gave encouraging results, showing only a marginal impairment of the immunoreactivity towards SW1222 cells for both the radiolabelled immunoconjugate batches. MALDI analysis of the immunoconjugates showed that, despite the excess of chelator used in the conjugation process, a modest chelator/antibody ratio of 0.6 was achieved when 10 molar equivalents of the THP^Me^-NCS were used. This is an average value, and likely includes immunoconjugate molecules with fewer and more chelators attached.

In view of its higher chelator/antibody ratio compared to the 5 molar equivalents batch, and its similar immunoreactivity, the 10 molar equivalents batch was used for a first pretargeting experiment. Based on our prior experience with the click chemistry-based pretargeting strategy [17], it was decided to use a 100 μg dose of the immunoconjugate. Unfortunately, neither in vivo imaging nor ex vivo biodistribution highlighted any significant difference between the pretargeted and the negative control (^68^Ga-only) group, suggesting that the tumour uptake first noticed in the pretargeting group was solely attributable to uptake of unchelated ^68^Ga in the tumour. Accumulation of gallium in some tumours is a known phenomenon, probably in part due to its similarity to Fe(III), which allows gallium to bind to transferrin and hence delivery to tissues overexpressing transferrin receptors [26]. This effect has been exploited in the past to image some types of cancer, as well as inflammation/infections, with ^67^Ga scintigraphy or SPECT [27]. The long-lived ^67^Ga isotope (in the form of ^67^Ga citrate) is preferred for these scans since a substantial target accumulation and contrast is only achieved after several hours or days. The use of ^68^Ga-citrate in PET imaging of lung and prostate cancer, and of bone infections, has been recently evaluated in clinical trials [28,29,30].

In an attempt to increase chelator concentration at the tumour site, a second pretargeting experiment was performed using a new immunoconjugate batch (30 molar equivalents) with an average chelator/antibody ratio of 1.0, the maximum achievable without partial precipitation of the immunoconjugate. This did not lead to any increase in the tumour uptake of ^68^Ga compared to the ^68^Ga-only control. On the other hand, a decreased uptake in bones was evident for this fourth group of animals and uptake in liver and large intestines was also reduced although to a lesser extent. Since all these organs are targets for accumulation of free gallium (especially bone), the decreased uptake suggests that part of the gallium may actually bind the antibody in vivo, but in circulation rather than at the tumour site. This effect could be exacerbated by the non-homogeneous chelator-to-antibody ratio of the immunoconjugate: although MALDI measurements gave a single average value, this is likely to represent a mixture of molecules with higher and lower ratios. Antibodies with more chelators attached are likely to have a lower immunoreactivity compared to those with a lower number of chelators and so are less likely to bind to their target antigen, but more likely to bind ^68^Ga. These immunoconjugate molecules could be radiolabelled while in circulation, diminishing ^68^Ga accumulation in bones/liver/intestine without significantly altering the tumour uptake, which is largely attributable to unchelated ^68^Ga.

Overall, no pretargeting-related tumour uptake was observed in these in vivo experiments and notably, no improvement in tumour uptake was visible in group 4 (DOL = 1) compared to group 2 (DOL = 0.6). If failure to see any pretargeted ^68^Ga uptake were a consequence of impaired affinity of the immunoconjugate, that would rule out the THP^Me^-NCS chelator for pretargeting purposes. If instead, this was due to an insufficient number of chelator molecules per antibody, no immediate solution presents itself, as attempted conjugation of more than one THP^Me^-NCS moiety per antibody resulted in precipitation of the immunoconjugate, likely due to aggregation. Therefore, we can conclude that the pretargeting via ^68^Ga chelation is not a viable option when using THP^Me^-NCS.

As a possible strategy to achieve higher DOL in future investigations, the use of the dendritic THP^Me^ system could be explored, to conjugate multiple chelators at a single point of attachment, thus increasing the concentration of chelator available at the tumour site [31].

Notably, although unsuccessful from a pretargeting point of view, these studies highlighted a significant amount of uptake of unchelated gallium in SW1222 tumours as early as 1–2 h after injection (≈13 %ID/g). This led us to evaluate the use of THP^Me^ as a blood clearance agent to increase the tumour-to-blood activity (and hence tumour-to-background) ratio. Notably, in this experiment THP^Me^ was able to clear ^68^Ga from the blood, leading to renal excretion, much more efficiently than from the tumours, resulting in a threefold enhancement of the tumour-to-blood activity ratio (shifting its value from <1 to >2) as calculated from the ex vivo biodistribution data at 3 h; ratios of tumour to other tissues were also enhanced. In agreement with these data, PET images of the mice before and after THP^Me^ treatment showed increased image contrast and better tumour visualisation. These results together suggest that the SW1222 colon carcinoma could be a good model to further investigate the use of unchelated ^68^Ga to image tumours with THP^Me^ as a blood-clearing agent. While THP^Me^ itself has not been evaluated in humans yet, one of its derivatives, THP^Me^-PSMA labelled with ^68^Ga, is in phase III clinical trials for imaging prostate cancer suggesting low toxicity of the THP^Me^ family and potential for clinical translation. The results also suggest that the hydroxypyridinone prototype deferiprone, an iron-chelating drug used safely in patients to treat iron overload, might likewise warrant evaluation as Ga^3+^ blood clearance agent as previously investigated [32].

Overall, the use of a Ga^3+^ blood-clearing agent could potentially be very useful in a clinical setting, especially for those countries relying on ^67^Ga-citrate imaging, allowing better contrast to be achieved, reducing time between radiotracer administration and imaging and perhaps allowing the tumour-targeting properties of gallium citrate to be exploited for PET imaging with ^68^Ga.

## 4. Materials and Methods

### 4.1. General Equipment and Consumables

THP^Me^-NH_2_ was obtained from Chematech (Dijon, France). THP^Me^ was synthesised according to published procedures [7]. The fully humanised IgG1 huA33 was obtained from the Ludwig Institute and the Olivia Newton-John Cancer Research Institute [33]. DFO-NCS was obtained from Macrocyclics Inc. (Plano, TX, USA) and the DFO-NCS-huA33 conjugate used for ^89^Zr radiolabelling was produced according to published procedures [17]. Unless otherwise stated, all other reagents were purchased from Sigma-Aldrich and used without further purification. All the buffers used for the huA33 experiments were prepared using Chelex-treated deionised water.

NMR spectra were acquired on a Bruker Advance 400 spectrometer (Bruker, Billerica, MA, USA) with a 5 mm QNP probe at 400.13 MHz, with chemical shifts referenced to the solvent peak. High resolution ESI-MS data were acquired on an Exactive Orbitrap Mass Spectrometer (Thermo Scientific, Waltham, MA, USA). Data were acquired and reference mass-corrected via a dual-spray electrospray ionisation source, using the factory-defined calibration procedure. Preparative HPLC was carried out using an Agilent Zorbax XDB-C18 column (21.2 × 150 mm, 5 µm) with a 5 mL/min flow rate and UV detection at 214 nm on an Agilent 1200 LC system (Agilent, Santa Clara, CA, USA). Mobile phase A was water with 0.1% trifluoroacetic acid (TFA), and mobile phase B was acetonitrile with 0.1% TFA. A gradient from 0% to 100% B (1%/min) was employed. ^68^Ga was obtained from Eckert and Ziegler generator, by elution with 5 mL of 0.1 M ultrapure HCl (Fluka-Honeywell, Charlotte, NC, USA). The eluate was collected in ≈1 mL fractions and the activity of all fractions was measured with a dose calibrator (Capintec, Florham Park, NJ, USA). The highest activity fraction was used for radiolabelling. ^89^Zr was produced at Memorial Sloan Kettering Cancer Center (MSKCC, New York, NY USA), through proton-beam bombardment of yttrium foil, and isolated as ^89^Zr-oxalate [34]. Uptake of radioactivity in SW1222 cells and organs was measured using a LKB Wallac 1282 Compugamma Gamma-counter (LKB, Vienna, Austria) at King’s College London (KCL), an Automatic Wizard gamma-counter (Perkin-Elmer, Waltham, MA, USA) at MSKCC.

### 4.2. Synthesis of THP^Me^-NCS

Bn-THP^Me^-NCS (now commercially available from Chematech, Dijon, France) was synthesised according to published procedures [13]. Briefly, *p*-phenylene-NCS (135 mg, 0.77 mmol) and DIPEA (75 μL, 0.44 mmol) were added to a solution of THP^Me^-NH_2_ (75 mg, 0.077 mmol) in DMF (2 mL) under stirring. After 15 min the DMF was removed under high-vacuum and the crude residue purified by preparative HPLC, yielding 43.7 mg of Bn-THP^Me^-NCS (0.035 mmol, 45% yield). Benzyl deprotection was then performed by addition of 5 mL of BCl_3_ solution (1 M in dichloromethane) and stirring under nitrogen followed by quenching with methanol on ice, solvent removal by rotary evaporation and purification via preparative HPLC to give 27.2 mg of THP^Me^-NCS (0.028 mmol, 80% yield). Product purity was verified by ^1^H NMR and high-resolution mass spectrometry.

^1^H NMR (400 Mhz, CD_3_OD) δ: 1.89 (t, 6H, **CH_2_**-CH_2_CONH-pyridinone), 2.14 (t, 6H, CH_2_-**CH_2_**-CONH-pyridinone), 2.40 (t, 2H, SCNH-CH_2_-**CH_2_**-CONH), 2.48 (s, 9H, 6-CH_3_), 3.83 (s broad: 9H, N-CH3, and 2H, SCNH-**CH_2_**-CH_2_-CONH), 4.59 (s, 6H, CONH-**CH_2_**-2), 6.86 (s, 3H, 5-H (pyridinone)), 7.12 (d, 2H, SCN-*m*Ph), 7.33 (d: 2 H, SCN-*o*Ph).

High. Res. ESI-MS: m/z for C_45_H_56_N_10_O_10_S_2_ [M+H]^+^ calc: 961.3695, found: 961.3684, [M+2H]^+^ calc: 481.1884, found: 481.1877. No other peaks were observed.

### 4.3. HuA33 Conjugation to THP^Me^-NCS

A solution of huA33 in PBS (7 μM, 0.8 mL, pH 7.4) was adjusted to pH 8.8–9.0 with a 0.1 M solution of Na_2_CO_3_, and 5, 10, 30 or 50 equivalents of THP^Me^-NCS (10 mM in DMSO) were added in small aliquots to prevent precipitation. The reaction mixture was shaken for 1 h at room temperature, then purified by size-exclusion chromatography on a PD-10 column (Sephadex G-25M PD-10 column, GE Healthcare, Chicago, IL, USA) eluting with ammonium acetate 0.2 M (for preliminary radiolabelling) or PBS (for preclinical experiments) and concentrated using centrifugal filtration units with a 50,000 molecular weight cut off (Amicon^TM^ ultra 2 mL, Millipore Corp, Burlington, MA, USA). The final concentration of the immunoconjugate was measured spectrophotometrically using a Biospec nano (Shimadzu, Kyoto, Japan), considering ε_280_ = 2.1 × 10^5^ L/mol·cm, and was used to determine the conjugation yield (Table S1).

### 4.4. MALDI-ToF Mass Spectrometry

THP^Me^-NCS-huA33 immunoconjugates were analysed by MALDI-ToF MS/MS using a Bruker ultraflex MALDI-ToF/ToF (Bruker Daltonic GmbH, Fällanden, Switzerland) according to the following procedure: 1 μL of each sample (1 mg/mL) was mixed with 1 μL of sinapic acid (10 mg/mL in 50% acetonitrile: water and 0.1% TFA). Then, 1 μL of the sample/matrix solution was spotted onto a stainless-steel target plate and allowed to dry. Ions were analysed in positive mode and external calibration was performed by use of a standard protein mixture (Bovine Serum Albumin). The measure was repeated three times for both the native antibody and each conjugation batch. The difference between the average mass of the immunoconjugate, as determined by a profile measurement of the sample and that of the native antibody (mode of distribution taken as an estimate of the mean) was divided by the exact mass of the chelator to determine the average number of chelators per antibody (DOL).

### 4.5. THP^Me^-NCS-HuA33 Radiolabelling

Radiolabelling of the immunoconjugate at a final concentration of 1 μM was performed in two different sets of conditions as described below. The radiolabelling mixture was incubated for 5 min at 37 °C. Success of the radiolabelling was verified by ITLC-SG (Pall Corp, New York, NY, USA) using 50 mM EDTA in water as a mobile phase and analysed on an AR-2000 ITLC plate reader (Bioscan Inc, Washington, DC, USA). The radiolabelling mixture was also applied to a PD10 column preconditioned with ammonium acetate 0.2 M (or PBS, depending on the radiolabelling mixture) to trap residual radiolabelled THP^Me^-NCS and scarcely soluble ^68^Ga species. The eluted fractions were analysed by ITLC and the residual activity trapped on the PD10 column was also measured.

Radiolabelling conditions tested:

1. pH 5.5–6.0 (verification of conjugation): 400 μL of the highest activity fraction (≈88 MBq) were mixed with 100 μL of ammonium acetate 4 M, and 42 μL of the neutralised solution (≈4 MBq) added to an immunconjugate solution (3 μL, 15 μM in ammonium acetate 0.2 M). Radiochemical yield (from ITLC): 95%; activity retained on PD10 column: 3%.
2. pH 7 (evaluation of the radiolabelling in the conditions required for the in vivo experiment): 410 μL of the highest activity fraction was adjusted to pH 7 using ammonium acetate (0.2 M, 400 μL) and sodium carbonate (0.1 M, 400 μL). Then, 40 μL of this solution (≈5 MBq) was added to 40 μL of immunoconjugate (2 μM in ammonium acetate 0.2 M or PBS). In a variation of the experiment, the effect of a 45 min delay between gallium eluate neutralisation and radiolabelling was also investigated. Radiochemical yield (from ITLC): 98 %; activity retained on PD10 column: 2.6% (3.0% when 45 min delay was applied).
3. A similar radiolabelling procedure was also applied to the native antibody (≈1 MBq, final antibody concentration = 7.5 μM) to verify that presence of chelator was necessary for successful radiolabelling. Radiochemical yield (from ITLC): 14%; activity retained on PD10 column: 97%.

### 4.6. DFO-NCS-huA33 Radiolabelling with^89^Zr

^89^Zr-oxalate (50 μL, 57 MBq), was diluted with 1 M oxalic acid to 100 μL, buffered to pH 7.5 using Na_2_CO_3_ 1M (106 μL) and added to 400 μL of DFO-NCS-huA33 solution in PBS (1.53 mg/mL, ≈10 μM). The radiolabelling mixture was agitated for 1 h at 37 °C. EDTA (50 mM solution in water) was added to reach a final concentration of 1 mM. ITLC analysis of the crude radiolabelling mixture (50 mM EDTA in water as a mobile phase) confirmed quantitative radiolabelling. The radiolabelling mixture was then applied to a PD10 column preconditioned with PBS. The resulting fractions were analysed by ITLC and the activity trapped on the column measured. The highest activity fractions were used to prepare doses for injections.

### 4.7. Cell Culture

Human colorectal cancer cell line SW1222 expressing the A33 antigen was obtained from Sigma-Aldrich and maintained in Minimum Essential Medium (Gibco^TM^, ThermoFisher, Waltham, MA, USA). Cell culture media were supplemented with 10% foetal calf serum, L-glutamine (2 mM), penicillin (100 units/mL), and streptomycin (0.1 mg/mL). Cell cultures were maintained at 37 °C in a humidified atmosphere of 5% CO_2_. A trypsin EDTA solution was used to passage the cells (0.25% trypsin/0.53 mM EDTA in Hank’s Buffered Salt Solution without calcium and magnesium).

### 4.8. Immunoreactive Fraction

An immunoreactivity assay was performed according to a modified literature procedure, measuring binding of ^68^Ga-THP^Me^-NCS-huA33 (5 and 10 equivalent batches) in excess antigen conditions, in which unmodified antibody would achieve quasi-quantitative target-binding [18,24]. Then, 10 μL of a radioimmunoconjugate solution in 0.4 ng/μL in PBS supplemented with 1% bovine serum albumin, ≈70 kBq, was added to a suspension of 2.0 × 10^7^ SW1222 colorectal cancer cells in 100 μL of medium. The resulting mixture was agitated by pipetting and incubated for 1 h on ice. Cells were then centrifuged (600 RCF for 5 min) and the supernatant carefully removed. The cells were then washed three times by resuspension of the pellet in 1 mL of ice-cold PBS followed by centrifugation and removal of the supernatant. Radioactivity in the cell pellet, supernatant and washings was measured by γ-counting. After background correction of the data, the immunoreactive fraction was calculated by dividing the counts in the cell pellet by the sum of the counts in the cell pellet, media and three washings and multiplying by 100. The experiment was performed in triplicate.

### 4.9. In Vivo Radiolabelling Studies in Healthy Mice

All in vivo experiments performed at KCL were carried out in accordance with UK Home Office regulations governing animal experimentation and complied with guidelines on responsibility in the use of animals in bioscience research of the UK Research Councils and Medical Research Charities, under UK Home Office project and personal licences.

Experiments included both PET/CT imaging and ex vivo biodistribution. BALB/c mice (female, 7–9 weeks, from Charles River, Wilmington, MA, USA) were used for clearance experiments in healthy animals. PET and CT scans were carried out on a nanoScan^®^ PET/CT (Mediso Medical Imaging Systems, Budapest, Hungary) [35] (PET: 1:5 coincidence, 5 ns time window, 400–600 KeV energy window, CT: 180 projection, 45 KVp). Respiration rate and bed temperature were monitored throughout all scans. PET/CT datasets were reconstructed using the Monte-Carlo based full 3D iterative algorithm Tera-Tomo (Mediso Medical Imaging Systems) [36] using 1:3 coincidence and a voxel size of 0.21 × 0.21 × 0.21 mm^3^. Images were analysed using VivoQuant software (inviCRO, London, UK).

Mice were anaesthetised with isoflurane (O_2_ flow rate: 1.0–1.5 L/min and isoflurane levels 2%–2.5%) and kept anaesthetised for the entire procedure so that dynamic PET scans could be performed. Activity was injected into the tail vein using an in-house made catheter (25 μL volume) or an insulin syringe (0.3 mL, Terumo, Tokyo, Japan), followed by injection of the chelator, or saline as a control, after a defined time interval. Relevant PET/CT scans were performed. Upon procedure completion, animals were sacrificed by neck dislocation while still anaesthetised, tissues harvested and weighed and ex vivo biodistribution assessed by γ-counting. Tubes containing different volumes of the radiotracer solution (corresponding to different %ID) were also counted as internal standards and the calibration curve obtained by linear regression of the data was used to convert measured CPM in %ID for every organ.

Ex vivo biodistribution was performed by collecting the whole organ in the case of tail, heart, lungs, liver, spleen, stomach and kidneys. The femur was used as representative of bone. Skin and fur were collected from the abdomen or the ears of the mouse and muscle was taken from the hind limb. Only part of the liver, small and large intestine was collected. All organs were washed in water to eliminate residual blood. Residual activity in the tail (due to any imperfections in IV injection) was subtracted from the total activity injected, and tail-corrected %ID was calculated for each organ. %ID/g was obtained by dividing the tail-corrected %ID of each organ by its weight.

### 4.10. THP^Me^ vs. DFO Clearance in Healthy Mice

This experiment was performed at KCL. BALB/c mice were anaesthetised and injected with ^68^Ga-acetate (100 µL, ≈10 MBq) via a tail vein injection, as previously described. After 1 h, 50 µL of a clearance agent solution (24 nmol of THP^Me^ or DFO, or saline as a control) was administered in the same way. At least one mouse per group was imaged before and after the second injection (PET from 20 to 45 min and from 80 to 105 min after injection of ^68^Ga-acetate). Mice were kept anaesthetised for the whole experiment, sacrificed at 130 min post ^68^Ga injection and ex vivo biodistribution performed.

### 4.11. Dose Finding Experiment

This experiment was performed at KCL. BALB/c mice were anaesthetised and injected with ^68^Ga-acetate (100 µL, 5–10 MBq) via a tail vein cannula. Then, 100 µL of the relevant THP^Me^ solution (0, 1, 5 or 20 µg) was administered in the same way 5 min after the first injection. All animals were kept under anaesthesia for the duration of the experiment. Mice were culled 15 min after the second injection and biodistribution performed.

### 4.12. Xenograft Model

All experiments in xenograft models were performed at MSKCC and were conducted according to the guidelines approved by the Research Animal Resource Center and Institutional Animal Care and Use Committee at MSK. Athymic nude female mice (Athymic Nude-nu, 8–10 weeks old) were obtained from Charles River Laboratories (Wilmington, MA, USA). Animals were housed in ventilated cages, given food and water ad libitum, and were allowed to acclimatise for approximately 1 week prior to inoculation of tumour cells. SW1222 tumours were induced on the left shoulder by a subcutaneous injection of 5 × 10^6^ cells suspended in 150 μL of a 1:1 mixture of fresh medium and BD Matrigel (BD Biosciences, Franklin Lakes, NJ, USA). The xenografts were monitored daily until an ideal size for imaging and biodistribution was reached (≈100–150 mm^3^) in 2–3 weeks.

### 4.13. PET Imaging of SW1222 Xenograft Models

Mice were randomly assigned to the five different experimental groups (for each group *n* = 5). Administration of antibody/radiotracers was performed via tail vein injection on conscious mice after gentle warming with a heat lamp. Approximately 5 min prior to imaging, mice were anaesthetised by inhalation of 2% isoflurane/oxygen gas mixture and positioned on the bed of a MicroPET Focus scanner (Concorde MicroSystem Inc, Knoxville, TN, USA). A 1% isoflurane anaesthesia was maintained throughout the scan. Static scans of 10 min each were recorded at different time points after injections. An energy window of 350–700 keV and a coincidence timing window of 6 ns were used. Data were sorted into 2-dimensional histograms by Fourier re-binning, and transverse images were reconstructed by filtered back-projection (FBP) into 0.87 × 0.87 × 0.8 mm^3^ matrix. The imaging data were normalised to correct for non-uniformity of PET response, dead-time count losses, positron branching ratio and physical decay to the time of injection. No attenuation, scatter or partial-volume averaging correction was applied. Activity concentrations (%ID/g) were calculated from the counting rates in the reconstructed images by use of a system calibration factor derived from the imaging of a mouse-sized water-equivalent phantom containing ^68^Ga or ^89^Zr. Images were analysed using ASIPro VMTM software (Concorde MicroSystems Inc, Knoxville, TN, USA). Mice were sacrificed by CO_2_ asphyxiation. Details of the different experiments performed are described below.

### 4.14. Ex Vivo Biodistribution

For all ^68^Ga experiments, tissues were harvested and weighed as described above and ex vivo biodistribution assessed by γ-counting. Calibration of the γ-counter with known amounts of activity allowed determination of the decay-corrected CPM/MBq conversion coefficient, which was used to calculate % injected dose (%ID) in each organ.

### 4.15. Imaging with ^89^Zr-DFO-NCS-huA33 (Positive Control)

Mice (group 1) were injected with ^89^Zr-DFO-NCS-huA33 in saline (200 μL, ≈7 MBq, 88 μg) and imaged at 25 h after injection. At 26 h post injection, mice were sacrificed.

### 4.16. Pretargeting Experiments

Mice were injected with 100 μg of THP^Me^-NCS-huA33 in saline using either the 10 eq. or the 30 eq. batch (groups 2 and 4, respectively; five mice per group). After 24 h, mice were injected with 8–10 MBq of ^68^Ga-acetate (pH 7) at the same time. PET imaging of the five mice was then performed starting from 50 to 90 min after injection (10 min per mouse) and then repeated 1 h later. Mice were sacrificed 3 h after ^68^Ga-acetate injection and ex vivo biodistribution performed.

### 4.17. ^68^Ga-Only Control

Mice (group 3) were injected with 8–10 MBq of ^68^Ga-acetate (pH 7) and imaging performed as described for the pretargeting experiments, from 1 to 2 h following administration of the radioactivity. Mice were culled at 3 h post injection and ex vivo biodistribution evaluated.

### 4.18. ^68^Ga Followed by THP^Me^ Blood Clearance

Mice (group 5) were injected with 10–11 MBq of ^68^Ga-acetate (pH 7). Imaging of two of the mice, one at 40 and one at 50 min after injection, was performed. A solution of THP^Me^ in saline (3 mg/mL) was prepared and pH adjusted to 7 with Na_2_CO_3_. A 400 μg/mL solution was prepared by dilution of the stock solution in saline. At 1 h after gallium injection, 20 μg of THP^Me^ (24 nmol, in 50 μL of saline) was also administered intravenously and all mice imaged between 20 and 60 min after THP^Me^ injection. Mice were sacrificed 3 h after the ^68^Ga injection and the ex vivo biodistribution of the activity was assessed.

### 4.19. Power Calculations

Sample size for in vivo experiments was calculated in order to have a statistical power (1-β) of 80% and a type I error probability (α) of 5%. A minimal sample size of *n* = 3 was calculated, to detect a difference between a signal-to-noise ratio of 10 ± 2 (experimental group) and 5 ± 2 (control) when performing one-way analysis of variances (ANOVA) followed by up to two pairwise comparisons between groups (THP^Me^ vs. DFO experiments). When three pairwise comparisons were considered (dose finding experiment) a minimal sample size of *n* = 4 was calculated.

### 4.20. Statistical Analysis of the Data

Data were analysed using GraphPad Prism, version 7.04 for Windows (GraphPad software). An unpaired, two-tailed Student’s *t*-test was generally used when comparing two groups. One-way ANOVA was used to analyse results where more than two groups were compared. In this case, a Tukey post-hoc test was employed to correct for multiple comparisons when performed between all the groups. A Dunnett post-hoc test was used for comparison between each of the experimental groups and the control group. Whenever variances of groups were found to be significantly different (according to an F test when comparing two groups, and to both Brown–Forsythe and Bartlett’s test when comparing more than two groups) the data were reanalysed using a Welch-adjusted *t*-test, which does not assume equal variances among groups.

## Figures and Tables

**Table 1:** Groups considered for in vivo pretargeting experiments.

Group	Mice N	Imaging Agent	Experimental Workflow
1	5	^89^Zr-DFO-NCS-huA33	*t* = 0 h, ^89^Zr-DFO-NCS-huA33 administration (88 μg, 7 MBq) *t* = 25 h, PET imaging
2	5	THP^Me^-NCS-huA33 (10 eq.) + ^68^Ga (Pretargeting)	*t* = 0 h, THP^Me^-NCS-huA33 administration (100 μg, 10 eq. batch) *t* = 24 h, acetate buffered ^68^Ga administration (8–10 MBq) *t* = 25 h, PET imaging
3	5	^68^Ga	*t* = 0 h, acetate buffered ^68^Ga administration (8–10 MBq) *t* = 1 h, PET imaging
4	5	THP^Me^-NCS-huA33 (30 eq.) + ^68^Ga (Pretargeting)	*t* = 0 h, THP^Me^-NCS-huA33 administration (100 μg, 30 eq. batch) *t* = 24 h, acetate buffered ^68^Ga administration (8–10 MBq) *t* = 25 h, PET imaging
5	5	^68^Ga + THP^Me^	*t* = 0 h, acetate buffered ^68^Ga administration (8–10 MBq) *t* = 1 h, THP^Me^ administration (20 μg) *t* = 1 h 20 min, PET imaging

## Supplementary Materials

Supplementary Information can be found at https://www.mdpi.com/1422-0067/21/4/1496/s1.

## Abbreviations

BFC	Bifunctional chelator
CPM	Counts per minute
CT	Computed tomography
DFO	Deferoxamine
DOL	Degree of labelling
ESI-MS	Electrospray ionisation mass spectrometry
HPLC	High pressure liquid chromatography
ID	Injected dose
ITLC	Instant thin layer chromatography
KCL	King’s College London
MALDI	Matrix-assisted laser desorption-ionisation
MIP	Maximum intensity projection
MSKCC	Memorial Sloan Kettering Cancer Center
PET	Positron emission tomography
SD	Standard deviation
TFA	Trifluoroacetic acid
THP	Tris(hydroxypyridinone)

## References

[1] Witzig T.E., Gordon L.I., Cabanillas F., Czuczman M.S., Emmanouilides C., Joyce R., Pohlman B.L., Bartlett N.L., Wiseman G.A., Padre N. (2002). Randomized controlled trial of yttrium-90-labeled ibritumomab tiuxetan radioimmunotherapy versus rituximab immunotherapy for patients with relapsed or refractory low-grade, follicular, or transformed B-cell non-Hodgkin’s lymphoma. J. Clin. Oncol..

[2] Kahn D., Williams R.D., Manyak M.J., Haseman M.K., Seldin D.W., Libertino J.A., Maguire R.T., Prostacint Study G. (1998). ^111^Indium-capromab pendetide in the evaluation of patients with residual or recurrent prostate cancer after radical prostatectomy. J. Urol..

[3] Bensch F., van der Veen E.L., Lub-de Hooge M.N., Jorritsma-Smit A., Boellaard R., Kok I.C., Oosting S.F., Schroder C.P., Hiltermann T.J.N., van der Wekken A.J. (2018). Zr-89-atezolizumab imaging as a non-invasive approach to assess clinical response to PD-L1 blockade in cancer. Nat. Med..

[4] Liu G.Z. (2018). A Revisit to the Pretargeting Concept-A Target Conversion. Front. Pharmacol..

[5] Verhoeven M., Seimbille Y., Dalm S.U. (2019). Therapeutic Applications of Pretargeting. Pharmaceutics.

[6] Altai M., Membreno R., Cook B., Tolmachev V., Zeglis B.M. (2017). Pretargeted Imaging and Therapy. J. Nucl. Med..

[7] Berry D.J., Ma Y., Ballinger J.R., Tavaré R., Koers A., Sunassee K., Zhou T., Nawaz S., Mullen G.E.D., Hider R.C. (2011). Efficient bifunctional gallium-68 chelators for positron emission tomography: Tris(hydroxypyridinone) ligands. Chem. Commun..

[8] Imberti C., Chen Y.L., Foley C.A., Ma M.T., Paterson B.M., Wang Y.F., Young J.D., Hider R.C., Blower P.J. (2019). Tuning the properties of tris(hydroxypyridinone) ligands: Efficient Ga-68 chelators for PET imaging. Dalton Trans..

[9] Imberti C., Nawaz S., Cooper M.S., Young J.D., Ma M.T., Berry D.J., Patterson B.M., Mullen G.E.D., Ballinger J.R., Blower P.J. (2015). Unusual gallium transchelation behavior of tris(hydroxypyridinone) chelators in plasma and in vivo. J. Nucl. Med..

[10] Nawaz S., Mullen G.E.D., Sunassee K., Bordoloi J., Blower P.J., Ballinger J.R. (2017). Simple, mild, one-step labelling of proteins with gallium-68 using a tris(hydroxypyridinone) bifunctional chelator: A Ga-68-THP-scFv targeting the prostate-specific membrane antigen. EJNMMI Res..

[11] Hofman M.S., Eu P., Jackson P., Hong E., Binns D., Iravani A., Murphy D., Mitchell C., Siva S., Hicks R.J. (2018). Cold Kit for Prostate-Specific Membrane Antigen (PSMA) PET Imaging: Phase 1 Study of Ga-68-Tris (Hydroxypyridinone)-PSMA PET/CT in Patients with Prostate Cancer. J. Nucl. Med..

[12] Young J.D., Abbate V., Imberti C., Meszaros L.K., Ma M.T., Terry S.Y.A., Hider R.C., Mullen G.E., Blower P.J. (2017). Ga-68-THP-PSMA: A PET Imaging Agent for Prostate Cancer Offering Rapid, Room-Temperature, 1-Step Kit-Based Radiolabeling. J. Nucl. Med..

[13] Ma M.T., Cullinane C., Imberti C., Baguna Torres J., Terry S.Y.A., Roselt P., Hicks R.J., Blower P.J. (2016). New Tris(hydroxypyridinone) Bifunctional Chelators Containing Isothiocyanate Groups Provide a Versatile Platform for Rapid One Step Labeling and PET Imaging with Ga-68^3+^. Bioconjug. Chem..

[14] Cusnir R., Imberti C., Hider R.C., Blower P.J., Ma M.T. (2017). Hydroxypyridinone Chelators: From Iron Scavenging to Radiopharmaceuticals for PET Imaging with Gallium-68. Int. J. Mol. Sci..

[15] GarinChesa P., Sakamoto J., Welt S., Real F.X., Rettig W.J., Old L.J. (1996). Organ-specific expression of the colon cancer antigen A33, a cell surface target for antibody-based therapy. Int. J. Oncol..

[16] Cook B.E., Adumeau P., Membreno R., Carnazza K.E., Brand C., Reiner T., Agnew B.J., Lewis J.S., Zeglis B.M. (2016). Pretargeted PET Imaging Using a Site-Specifically Labeled Immunoconjugate. Bioconjug. Chem..

[17] Zeglis B.M., Sevak K.K., Reiner T., Mohindra P., Carlin S.D., Zanzonico P., Weissleder R., Lewis J.S. (2013). A Pretargeted PET Imaging Strategy Based on Bioorthogonal Diels-Alder Click Chemistry. J. Nucl. Med..

[18] Adumeau P., Carnazza K.E., Brand C., Carlin S.D., Reiner T., Agnew B.J., Lewis J.S., Zeglis B.M. (2016). A Pretargeted Approach for the Multimodal PET/NIRF Imaging of Colorectal Cancer. Theranostics.

[19] Moeschlin S., Schnider U. (1963). Treatment of primary and secondary hemochromatosis and acute iron poisoning with a new, potent iron-eliminating agent (Desferrioxamine-B). N. Engl. J. Med..

[20] Hoffer P.B., Samuel A., Bushberg J.T., Thakur M. (1979). Desferoxamine mesylate (Desferal)—Contrast-enhancing agent for Ga-67 imaging. Radiology.

[21] Koizumi K., Tonami N., Hisada K. (1982). Deferoxamine mesylate enhancement of Ga-67 tumor-to-blood ratios and tumor imaging. Eur. J. Nucl. Med..

[22] Tsionou M.I., Knapp C.E., Foley C.A., Munteanu C.R., Cakebread A., Imberti C., Eykyn T.R., Young J.D., Paterson B.M., Blower P.J. (2017). Comparison of macrocyclic and acyclic chelators for gallium-68 radiolabelling. RSC Adv..

[23] Cooper M.S., Sabbah E., Mather S.J. (2006). Conjugation of chelating agents to proteins and radiolabeling with trivalent metallic isotopes. Nat. Protoc..

[24] McDevitt M.R., Finn R.D., Ma D., Larson S.M., Scheinberg D.A. (1999). Preparation of alpha-emitting Bi-213-labeled antibody constructs for clinical use. J. Nucl. Med..

[25] Sephton R.G., Hodgson G.S., Deabrew S., Harris A.W. (1978). Ga-67 and Fe-59 distributions in mice. J. Nucl. Med..

[26] Chitambar C.R. (2016). Gallium and its competing roles with iron in biological systems. Biochim. Biophys. Acta Mol. Cell Res..

[27] Larson S.M., Milder M.S., Johnston G.S. (1973). Interpretation of Ga-67 photoscan. J. Nucl. Med..

[28] Behr S.C., Aggarwal R., Seo Y., Aparici C.M., Chang E., Gao K.T., Tao D.H., Small E.J., Evans M.J. (2016). A Feasibility Study Showing Ga-68 Citrate PET Detects Prostate Cancer. Mol. Imaging Biol..

[29] Vorster M., Maes A., Jacobs A., Malefahlo S., Pottel H., Van de Wiele C., Sathekge M.M. (2014). Evaluating the possible role of Ga-68-citrate PET/CT in the characterization of indeterminate lung lesions. Ann. Nucl. Med..

[30] Nanni C., Errani C., Boriani L., Fantini L., Ambrosini V., Boschi S., Rubello D., Pettinato C., Mercuri M., Gasbarrini A. (2010). Ga-68-Citrate PET/CT for Evaluating Patients with Infections of the Bone: Preliminary Results. J. Nucl. Med..

[31] Imberti C., Terry S.Y.A., Cullinane C., Clarke F., Cornish G.H., Ramakrishnan N.K., Roselt P., Cope A.P., Hicks R.J., Blower P.J. (2017). Enhancing PET Signal at Target Tissue in Vivo: Dendritic and Multimeric Tris(hydroxypyridinone) Conjugates for Molecular Imaging of α_v_β_3_ Integrin Expression with Gallium-68. Bioconjug. Chem..

[32] Eybl V., Svihovcova P., Koutensky J., Kontoghiorghes G.J. (1992). Interaction of L1, L1NAII and deferoxamine with gallium in vivo. Drugs Today.

[33] King D.J., Antoniw P., Owens R.J., Adair J.R., Haines A.M.R., Farnsworth A.P.H., Finney H., Lawson A.D.G., Lyons A., Baker T.S. (1995). Preparation and preclinical evaluation of humanized A33 immunoconjugates for radioimmunotherapy. Br. J. Cancer.

[34] Holland J.P., Sheh Y., Lewis J.S. (2009). Standardized methods for the production of high specific-activity zirconium-89. Nucl. Med. Biol..

[35] Szanda I., Mackewn J., Patay G., Major P., Sunassee K., Mullen G.E., Nemeth G., Haemisch Y., Blower P.J., Marsden P.K. (2011). National Electrical Manufacturers Association NU-4 Performance Evaluation of the PET Component of the NanoPET/CT Preclinical PET/CT Scanner. J. Nucl. Med..

[36] Magdics M., Szirmay-Kalos L., Toth B., Legrady D., Cserkaszky A., Balkay L., Domonkos B., Voelgyes D., Patay G., Major P. Performance Evaluation of Scatter Modeling of the GPU-based “Tera-Tomo” 3D PET Reconstruction. Proceedings of the 2011 IEEE Nuclear Science Symposium Conference Record.

