# Supplementary material for: Manipulating the In Vivo Behaviour of 68Ga with Tris(Hydroxypyridinone) Chelators: Pretargeting and Blood Clearance
Source: Int J Mol Sci. 2020 Feb 22;21(4):1496. doi: 10.3390/ijms21041496 (PMC7073083; doi:10.3390/ijms21041496)
Supplement: Supplementary file 1 [file ijms-21-01496-s001.pdf]

Electronic Supplementary Information

Manipulating the *in vivo* behaviour of ^{68}Ga with tris(hydroxypyridinone) chelators: pretargeting and blood clearance.

Content

THP^{Me} changes ^{68}Ga biodistribution *in vivo*

Figure S1 ^{68}Ga biodistribution in mice treated with THP^{Me}/DFO 1h post ^{68}Ga administration

Figure S2 ^{68}Ga biodistribution in mice treated with different doses of THP^{Me} 5 minutes post ^{68}Ga administration

Immunoconjugate characterisation and radiolabelling

Table S1 Immunoconjugation data

Figure S3 MALDI mass spectra

Figure S4 ^{68}Ga radiolabelling of THP^{Me}-NCS-huA33 vs huA33

Figure S5 ^{68}Ga radiolabelling of THP^{Me}-NCS-huA33, 45 minutes after neutralisation

Figure S6 ^{89}Zr radiolabelling of huA33-DFO

***In vivo* pretargeting studies**

Figure S7 PET images of ^{68}Ga in a SW1222 model pretargeted with THP^{Me}-NCS-huA33 (group 2, 10 eq. batch)

Figure S8 PET images of ^{68}Ga in a SW1222 model (group 3, negative control)

Figure S9 PET images of ^{68}Ga in a SW1222 model pretargeted with THP^{Me}-NCS-huA33 (group 4, 30 eq. batch)

Figure S10 ^{68}Ga biodistribution in mice pretargeted with THP^{Me}-NCS-huA33 vs negative control

Table S2. Comparison between mean %ID/g and tumour/organ ratio for ^{68}Ga and ^{68}Ga + THP^{Me} groups

THP^{Me} changes ^{68}Ga biodistribution *in vivo*

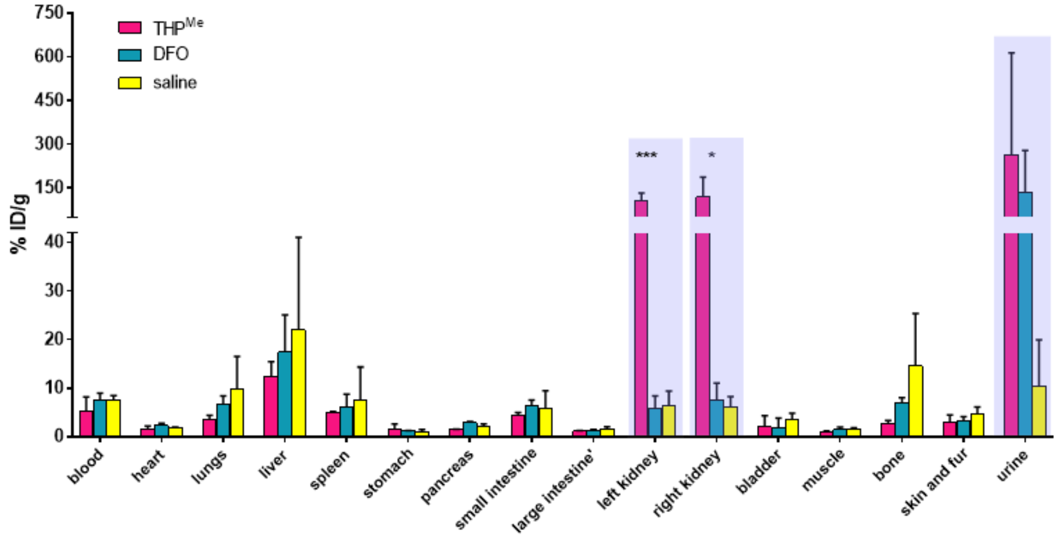

Figure S1. *Biodistribution as determined by ex vivo organ counting of ^{68}Ga (130 min after ^{68}Ga injection) in mice treated with ^{68}Ga-acetate followed at 1 hour post injection by administration of a blood clearance agent (THP^{Me} or DFO, 24 nmol) or saline. Values are expressed as mean ±SD, N = 3. Statistical analysis was performed for each organ using ANOVA followed by Dunnett post-hoc comparison between each experimental group and the saline control (black *). For the groups shaded in blue, unequal variances were obtained (Brown-Forsythe and Bartlett test) and therefore Welch-adjusted t-test was also carried out (blue *). P values for each blood clearance agent are evaluated in comparison with the negative control group (* = $P \leq 0.05$, *** = $P \leq 0.0001$).*

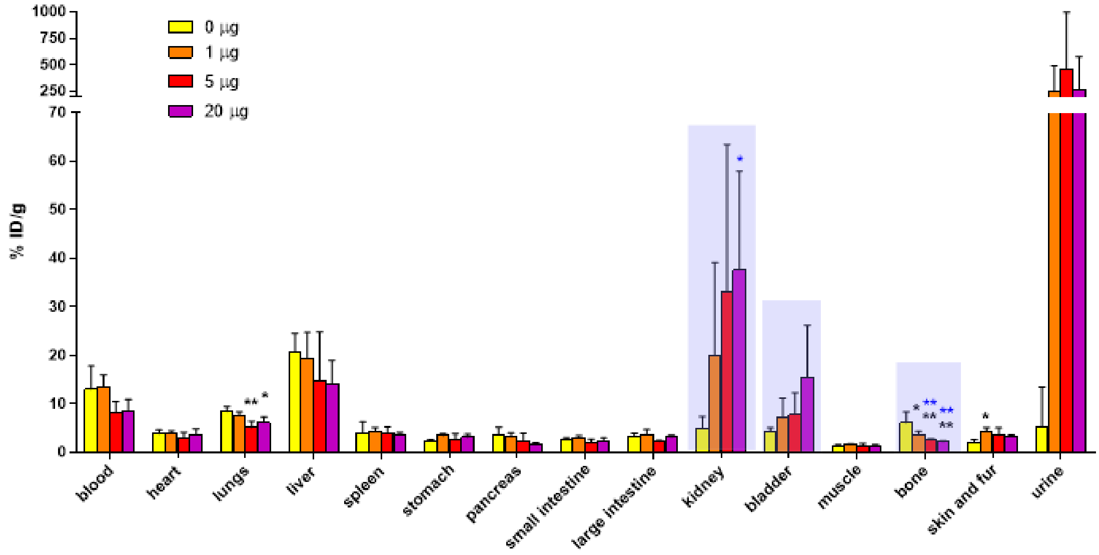

Figure S2. *Ex vivo biodistribution of ^{68}Ga (20 min after ^{68}Ga injection) in mice treated with different doses of THP^{Me}, with a delay of 5 min between the two injections. Values are expressed as mean ± SD (N = 4 except for urine where N = 3 for 0, 1 and 5 µg). Statistical analysis was performed for each organ using one-way ANOVA, followed by Dunnett post-hoc test to correct for multiple comparisons (black *). For the groups shaded in blue, unequal variances were obtained (Brown-Forsythe and Bartlett test) and Welch-adjusted t-test was also carried out (blue *). P values for a given concentration are evaluated in comparison with the relevant 0 µg value (* = $P \leq 0.05$, ** = $P \leq 0.01$).*

Immunoconjugate characterisation and radiolabelling

Table S1. *Summary of characterisation data for the different THP^{Me}-NCS-huA33 conjugates. Batches used for in vivo experiment are highlighted in pink. Immunoreactive fraction data are reported as average ± SD. *Radiolabelling of the 50 equivalents batch was not performed since precipitation occurred during overnight storage in the fridge.*

Immunoconjugate batch	Conjugation Yield	Radiolabelling at 1 µM	Immunoreactive fraction	Number of chelators/antibody
**5 eq**	85.7 %	quantitative	92.7 ± 0.7	0.4
**10 eq**	85.9 %	quantitative	89.2 ± 3.1	0.6
**30 eq**	85.3 %	quantitative	N/A	1
**50 eq**	92.4 %	*	N/A	N/A

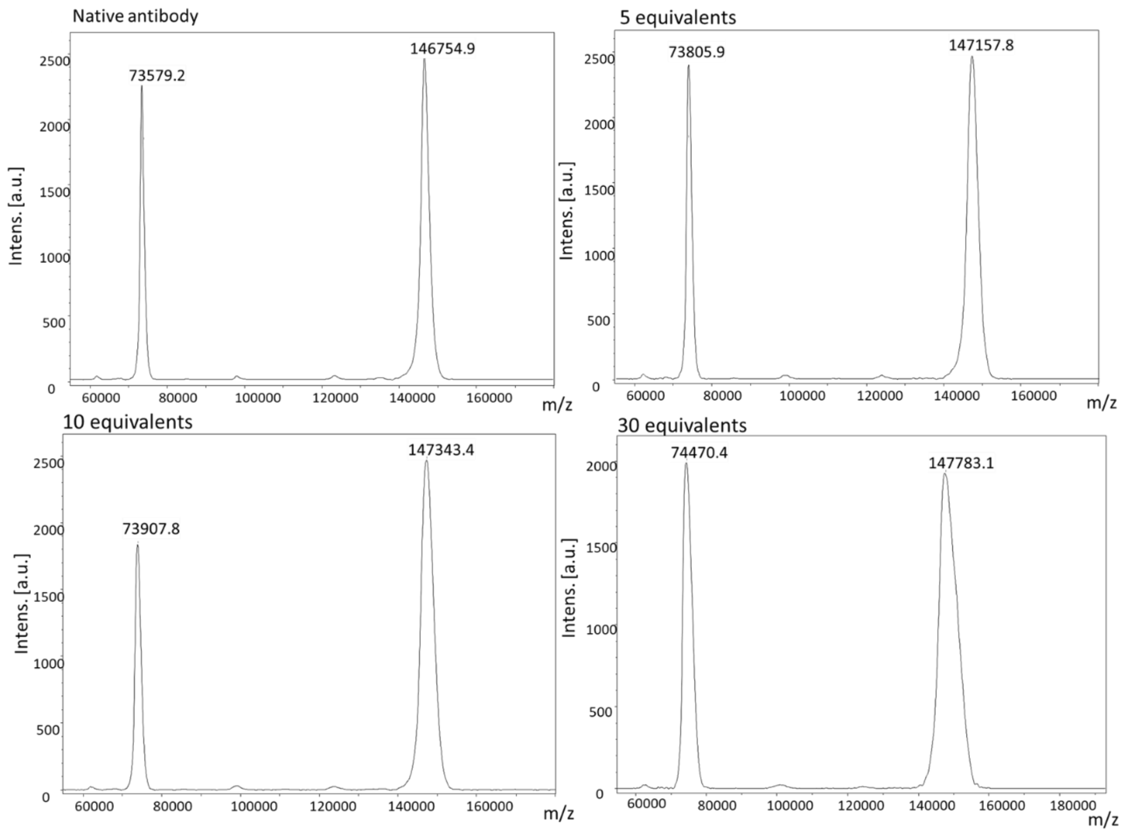

Figure S3. *Examples of MALDI mass spectra for the native antibody and different batches of immunoconjugate. The data are presented as profile m/z spectra, showing both $[M]^+$ and $[M]^{2+}$ species. The reported m/z values represent the average mass of the immunoconjugate in the sample.*

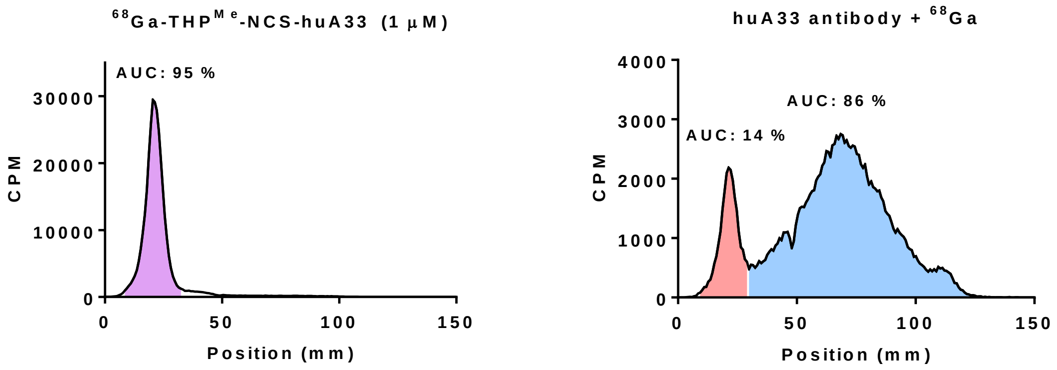

Figure S4. *ITLC profiles of the crude radiolabelling mixture for the THP^{Me}-huA33conjugate (left) and the native huA33 antibody (right). In the mobile phase utilised, the radiolabelled antibody stays at the baseline of the ITLC while any unchelated metal smears throughout the TLC strip. For every peak, the area under the curve (AUC) has been calculated and reported as a percentage. Notably, while quantitative radiolabelling is observed for the immunoconjugate, the native antibody has only 14 % of the radioactivity associated. Purification on a size-exclusion column resulted in only 3 % of the activity eluted in the void volume for the native antibody suggesting that most of the ^{68}Ga was either non-chelated or only loosely bound. On the contrary, 97.5 % of the activity was eluted in the column void volume for the THP^{Me}-NCS-huA33 immunoconjugate, confirming quantitative radiolabelling.*

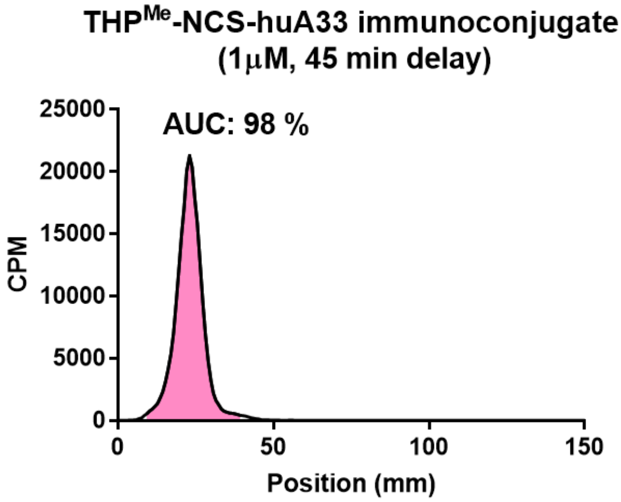

Figure S5*. ITLC profile and AUC for THP^{Me}-NCS-huA33 radiolabelling (1 μM) at pH 7, with 45 min delay between neutralisation of the ^{68}Ga eluate and radiolabelling of the immunoconjugate.*

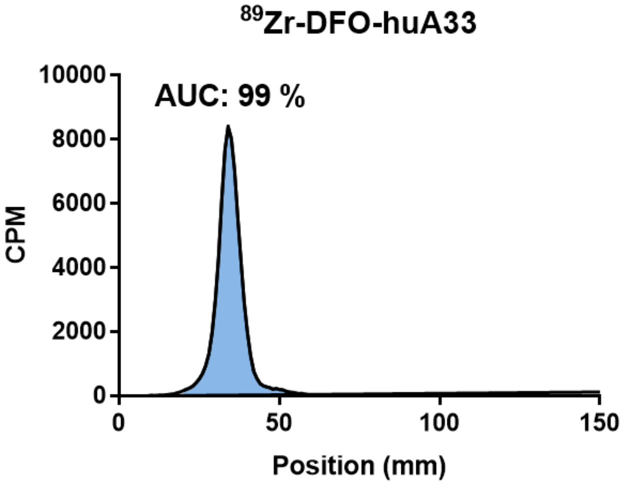

Figure S6. *ITLC profile and AUC for huA33-DFO (6.7 μM) radiolabelling with ^{89}Zr at pH 7.5 (fraction 2).*

In vivo Pretargeting studies

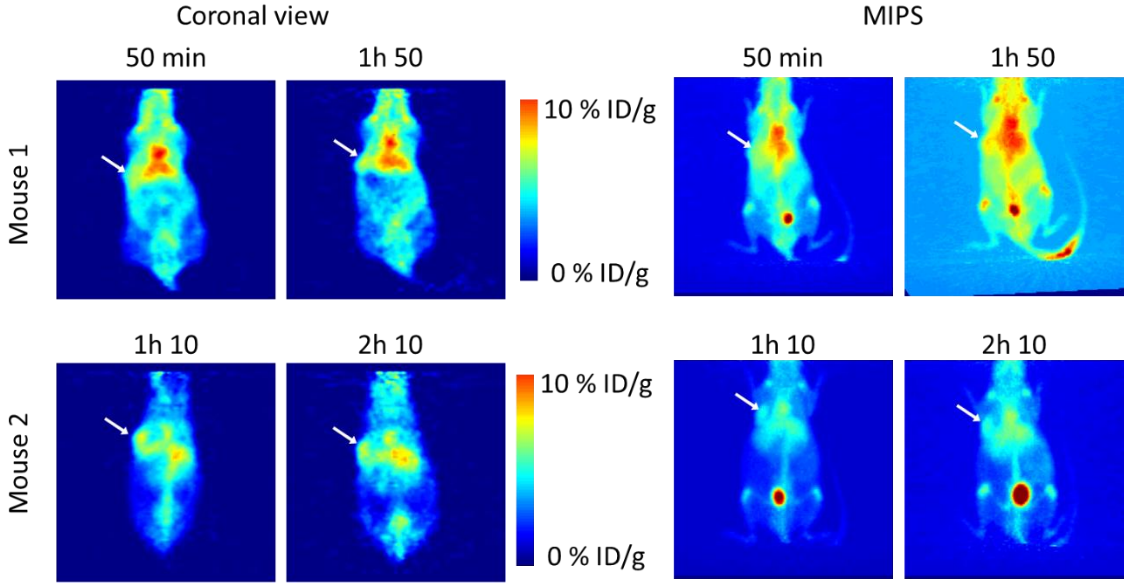

Figure S7. *PET images showing the biodistribution of ^{68}Ga in nude mice bearing SW1222 xenografts, pretreated with THP^{Me}-NCS-huA33 (10 equivalents batch, group 2) 24 hours before ^{68}Ga injection. For each mouse two scans of 10 min each were performed at ≈ 1 and 2 h post ^{68}Ga injection. Images are reported as coronal view (left panel) and MIP (right panel). White arrows indicate location of the tumour.*

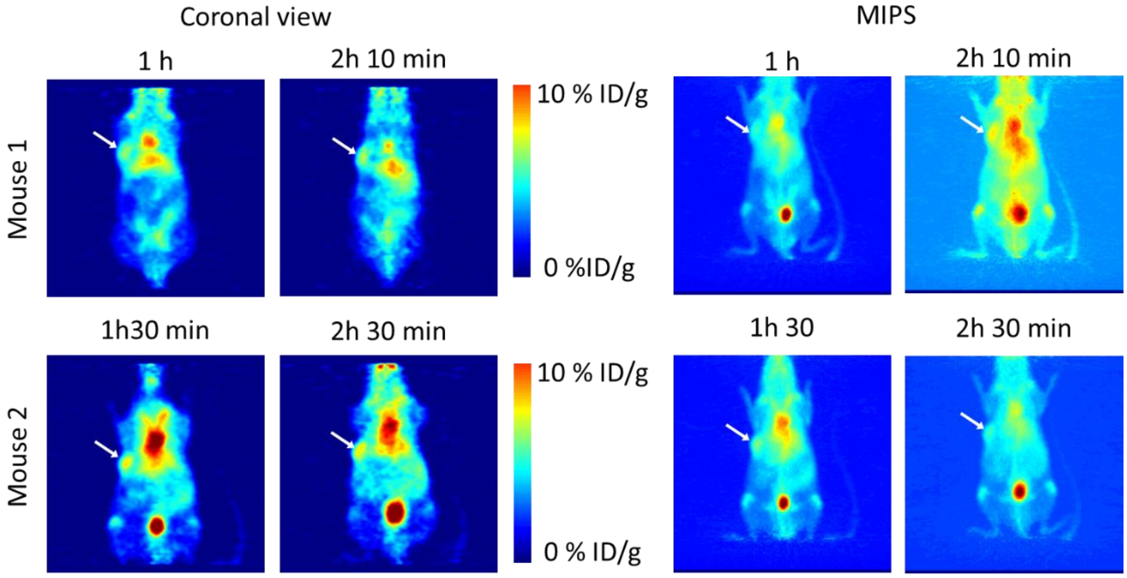

Figure S8. *PET images illustrating the biodistribution of ^{68}Ga in nude mice bearing SW1222 xenograft, without antibody conjugate preinjection, at ≈ 1 and 2 h after ^{68}Ga injection (group 3). For each mouse coronal view (left panel) and MIP (right panel) are reported. White arrows indicate location of the tumour.*

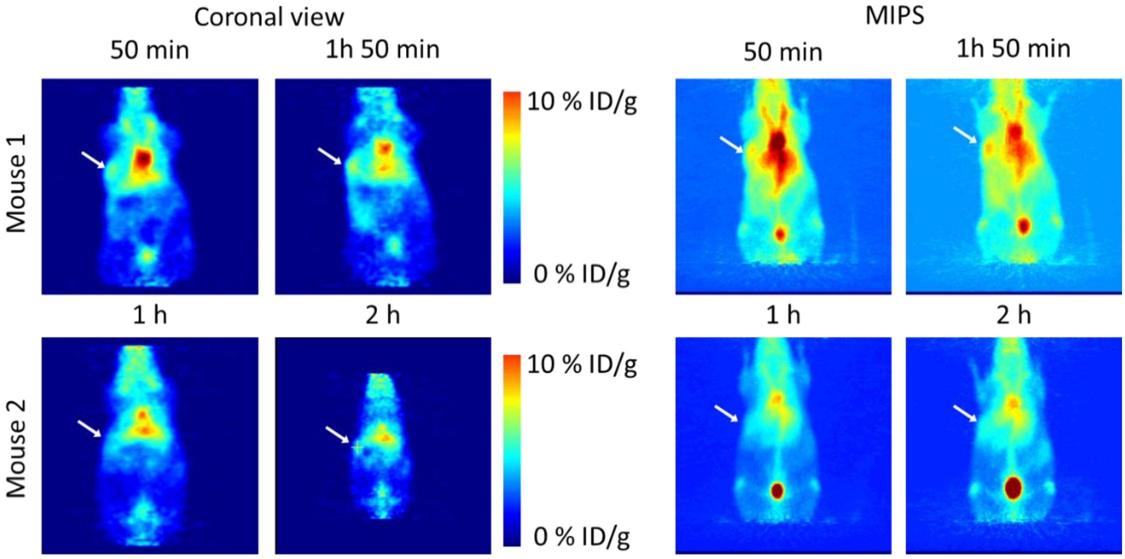

Figure S9. *PET images displaying the biodistribution of* ^{68}*Ga in nude mice bearing SW1222 xenografts, pretreated with THP*^{Me}*-NCS-huA33 (30 equivalents batch, group 4) 24 h before* ^{68}*Ga administration. Scans performed at ≈ 1 and 2 h after* ^{68}*Ga injections are reported as coronal view (left panel) and MIP (right panel). White arrows indicate location of the tumour.*

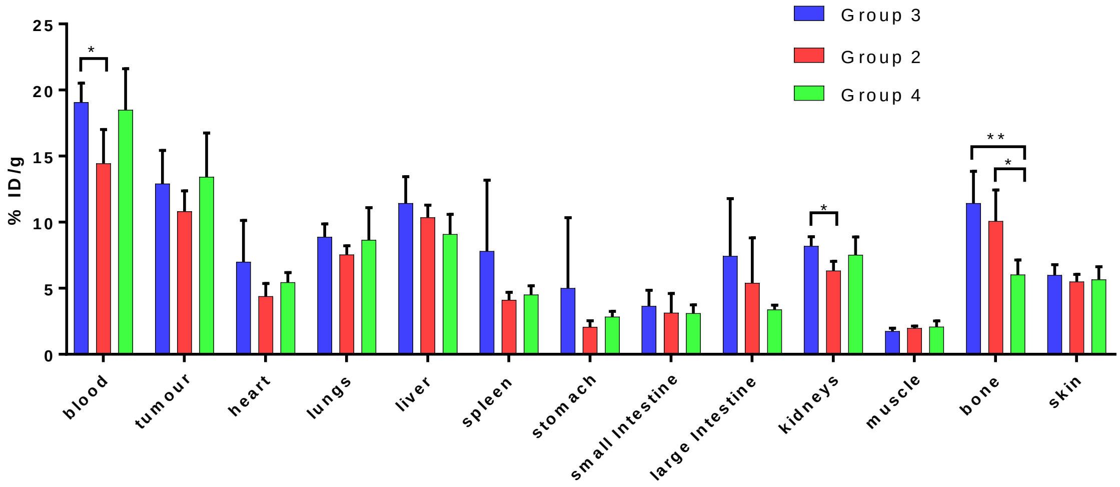

Figure S10. *Ex vivo* ^{68}*Ga biodistribution in nude mice bearing SW1222 xenografts at 3 hours after* ^{68}*Ga injections, comparing mice pretreated with THP*^{Me} *-NCS-huA33 24 hours before* ^{68}*Ga administration (10 equivalents batch - group 2 in red, and 30 equivalents batch - group 4 in green), and mice that were not pretreated with any immunoconjugate (group 3, blue). For each group N = 5, data are presented as mean ± SD.*

Table S2. *Comparison between mean %ID/g and tumour/organ ratio for groups 3 (^{68}Ga) and 5 (^{68}Ga followed by THP^{Me}). P values resulting from a t-test comparing the two groups' mean for each organ are also reported.*

	P value	Group 3		Group 5	
		Mean	Mean tumour/organ	Mean	Mean tumour/organ
**Blood**	5.71E-09	19.08	0.7	1.75	2.3
**Tumour**	6.29E-05	12.91	1.0	3.97	1.0
**Heart**	3.87E-07	5.36	2.4	0.66	6.1
**Lungs**	2.01E-07	8.83	1.5	1.20	3.3
**Liver**	1.43E-05	10.30	1.3	3.52	1.2
**Spleen**	9.45E-06	5.21	2.5	1.63	2.4
**Stomach**	0.004019	2.42	5.9	0.89	4.6
**Small intestine**	0.003237	3.66	4.3	1.31	3.3
**Large intestine**	0.07185	7.45	1.9	3.40	1.2
**Kidneys**	0.000557	8.19	1.6	4.21	1.1
**Muscle**	6.37E-07	1.76	7.4	0.25	16.9
**Bone**	2.07E-05	11.43	1.2	1.81	2.2
**Skin**	6.5E-07	5.99	2.2	0.86	4.7
